# Bioindicators of severe ocean acidification are absent from the end-Permian mass extinction

**DOI:** 10.1038/s41598-022-04991-9

**Published:** 2022-01-24

**Authors:** William J. Foster, J. A. Hirtz, C. Farrell, M. Reistroffer, R. J. Twitchett, R. C. Martindale

**Affiliations:** 1 Institut für Geologie, Centrum für Erdsystemforschung und Nachhaltigkeit, Universität Hamburg, Hamburg, Germany; 2 University of Texas at Austin, Jackson School of Geosciences, Austin, TX USA; 3 School of Earth Sciences, University College Dublin, Dublin, Ireland; 4 Department of Earth Sciences, Natural History Museum, London, SW7 5BD UK

**Keywords:** Palaeontology, Climate change

## Abstract

The role of ocean acidification in the end-Permian mass extinction is highly controversial with conflicting hypotheses relating to its timing and extent. Observations and experiments on living molluscs demonstrate that those inhabiting acidic settings exhibit characteristic morphological deformities and disordered shell ultrastructures. These deformities should be recognisable in the fossil record, and provide a robust palaeo-proxy for severe ocean acidification. Here, we use fossils of originally aragonitic invertebrates to test whether ocean acidification occurred during the Permian–Triassic transition. Our results show that we can reject a hypothesised worldwide basal Triassic ocean acidification event owing to the absence of deformities and repair marks on bivalves and gastropods from the Triassic *Hindeodus parvus* Conodont Zone. We could not, however, utilise this proxy to test the role of a hypothesised acidification event just prior to and/or during the mass extinction event. If ocean acidification did develop during the mass extinction event, then it most likely only occurred in the latest Permian, and was not severe enough to impact calcification.

## Introduction

One catastrophic consequence of a rapid release of carbon dioxide (CO_2_) into the atmosphere is the increased uptake of CO_2_ into the oceans and the development of ocean acidification, which is detrimental to marine organisms. Ocean acidification is a complicated phenomenon associated with a decrease in ocean pH, shifts in carbonate speciation, and the calcium carbonate saturation state (Ω)^1^. Furthermore, unlike ocean deoxygenation, which becomes more severe with a progressive rise in temperature, carbonate undersaturation can only develop if the uptake of CO_2_ into the oceans is relatively rapid, and subsequently can only persist for a few tens of thousands of years^2^. This has made understanding the role, timing, duration and extent of ocean acidification in past hyperthermals problematic, especially because geoscientists lack a robust and widely accepted proxy for ocean acidification that can be applied beyond the Cenozoic. This controversy could not be greater than for the end-Permian mass extinction event, which occurred ~ 252 million years ago, just prior to the Permian/Triassic boundary^3^, and wiped out ~ 74% of marine genera. It is generally accepted that this event was caused by climate change, following rapid release of CO_2_ into the atmosphere as a result of the eruptions of the Siberian Traps large igneous province^4,5^.

Permian–Triassic ocean acidification is controversial because different studies, using different proxies and approaches, have provided conflicting and mutually exclusive hypotheses related to its supposed timing and duration. Inferences from earth-system models^6^, sedimentology^7^, and extinction selectivity^8^ have suggested that ocean acidification, if present, would only have occurred between the mass extinction event and the Permian/Triassic boundary. On the other hand, a negative calcium isotope (δ^44/40^Ca) excursion has been used to infer an ocean acidification event during the post-extinction, basal-Triassic *Hindeodus parvus* Conodont Zone only^9–11^. In contrast, Boron isotopes (δ^11^B), from bulk rock samples indicate that pH did not decline until even later, in the *Isarcicella isarcica* Conodont Zone, and also suggest that oceans became more alkaline during the mass extinction and across the P/Tr boundary^12^. Analyses of δ^11^B from brachiopod calcite indicate instead that a decrease in pH started with the mass extinction event and continued into the basal Triassic, in agreement with earth-system modelling^13^, but this new data is confounded with potential issues of diagenetic alteration. Ocean acidification and carbonate undersaturation in the basal Triassic are not, however, supported by the sedimentological record of the *H. parvus* Zone, e.g., the widespread development of anachronistic carbonate facies^14^, which indicates conditions that were at least saturated, rather than undersaturated, and supports the bulk rock δ^11^B results. This means that there are three different hypotheses for the timing of ocean acidification during the Permian–Triassic climate crisis: H1—at the onset of the end-Permian mass extinction event, H2—during the *H. parvus* Zone, and H3—during the *I. isarcica* Zone.

An alternative approach to test the timing and presence of an ocean acidification event is to use evidence directly from the animals which were inhabiting marine systems at that time. Observations of the in vivo responses of invertebrates to experimentally lowered pH and to naturally acidic habitats, such as those associated with submarine volcanoes and seeps (local hypercapnia and low pH), show a myriad of negative effects (e.g., Ref.^15^). Changes that permanently alter the preservable parts of invertebrates, such as the shell, should be readily recorded in the fossil record; these include reduced growth rates, smaller sizes, increased porosity, shell degradation, disordered calcite fibre ornamentation, morphological deformities, increased dissolution, and shell repair ^15–23^.

In this study, we investigated fossilised shells of gastropods and bivalves for dissolution and repair marks to test hypotheses of past ocean acidification. Repair marks in particular provide unequivocal evidence that the individual animal lived in acidic waters for part or all of its life. Importantly, the pattern of shell repair following in vivo dissolution is unique to ocean acidification, and different to, for example, repair from attempted predation or physical breakage^19^ (Supplementary Table S1). Dissolution and repair marks also lead to morphological deformities (i.e., mechanical damage to the shell), which can be distinguished from morphological malformations (i.e., abnormal development, typically caused by other environmental stressors) in well-preserved fossils. This is because deformities occur alongside shell damage, whereas malformations occur in the absence of damage to the shell. Post-mortem dissolution can be distinguished by the lack of associated repair, impact on growth lines, and by its position, e.g. on the internal surfaces of the shell that would not be exposed to seawater during the life of the animal. Several studies have utilised pteropods (a group of aragonitic, planktonic gastropods) as bioindicators of ocean acidification during the Cenozoic (e.g., Ref.^24^), but the application of other invertebrate groups to investigate ocean acidification in the Mesozoic or Palaeozoic has not previously been undertaken.

**Figure 1:**
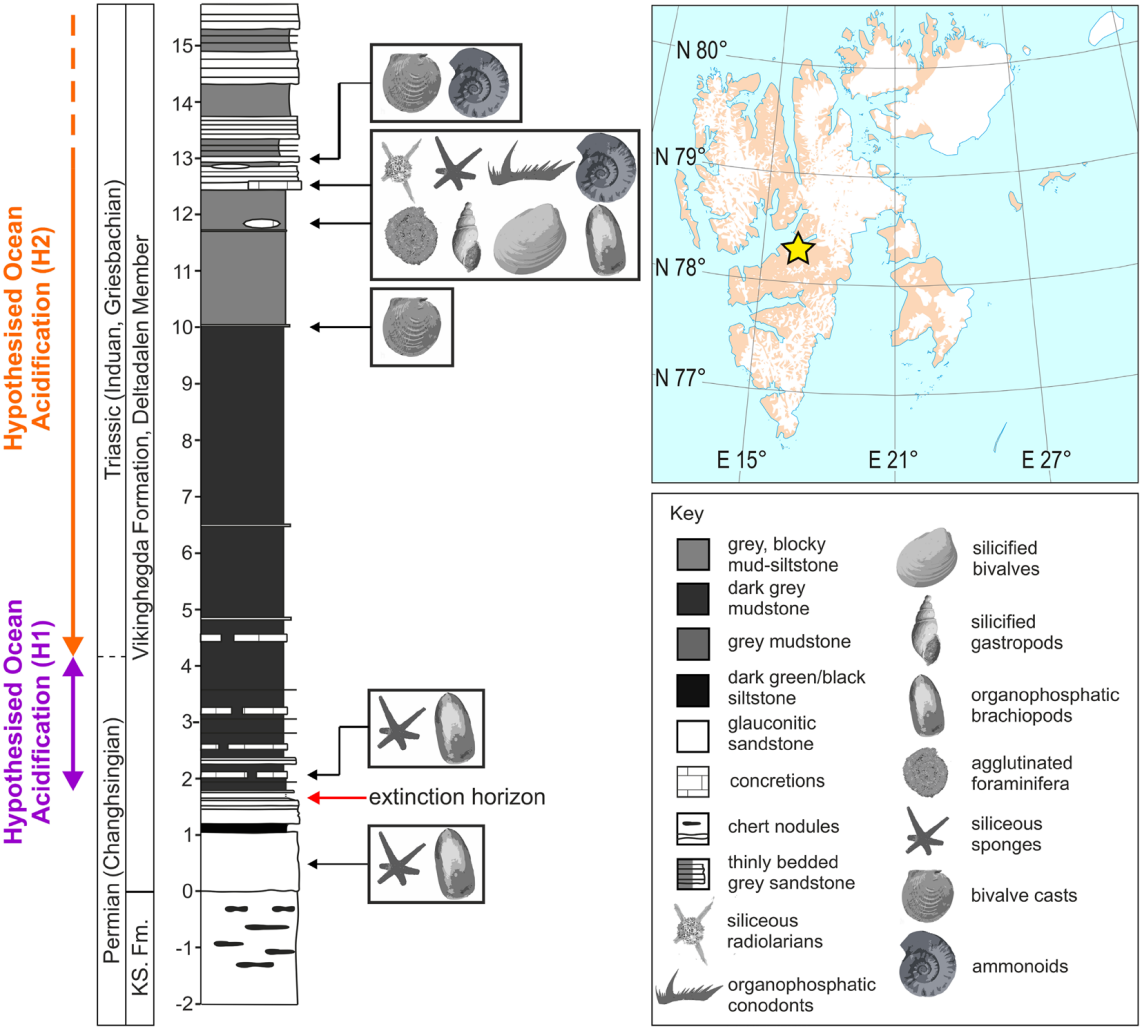
Stratigraphic succession of the Permian–Triassic transition at Lusitaniadalen, Svalbard. Inset shows the location of the studied section (marked by the star). The samples that yielded fossils are shown with the main fossil groups and their shell mineralogy. The two hypothesised ocean acidification intervals being tested in this study are shown to the left of the stratigraphic log. The position of the Permian/Triassic boundary is based on the occurrence of *H. parvus* in the nearby Deltadalen section following Zuchuat et al.^26^. KS = Kapp Starostin Formation. Note: 0 m marks the base of the Vikinghøgda Formation and the extinction horizon is inferred from the disappearance of intense bioturbation, following Nabbefeld et al.^27^.The inset map is a sketch map based on https://toposvalbard.npolar.no/.

Well-preserved fossil specimens are required to identify morphological deformities and very few locations exhibit high-quality preservation through the Permian–Triassic interval^25^. We will, therefore, focus on the fossil record from the Permian–Triassic succession exposed at Lusitaniadalen, Svalbard (Fig. 1), which records the best-preserved fossils known from this critical interval as well as continuous deposition during the mass extinction event^25^. Furthermore﻿, this fossil assemblage includes bivalves and gastropods that constructed their shells from aragonite and possessed a planktotrophic larval stage, enabling us to test for the presence of acidic conditions at different water depths and at different times in ontogeny. The most recent stratigraphic framework for this succession^26^ also allows us to test two of the hypothesised ocean acidification events (H1 and H2), i.e., at the extinction horizon and in the *H. parvus* Zone (Fig. 1).

## Results

### Faunal composition changes

Two different faunal communities are recognised through the Permian–Triassic interval at Lusitaniadalen. Prior to the mass extinction event, assemblages are dominated by the organophoshatic brachiopod *Lingularia freboldi* and sponge spicules from a glauconitic sandstone, which records a species turnover to *Lingularia* sp. and the presence of abundant siliceous sponge spicules across the mass extinction event. This minor faunal turnover corresponds to the first hypothesised ocean acidification interval (Fig. 1). Ecologically, the major changes across the extinction horizon at this locality are a decrease in the shell size of *Lingularia* and a reduction in the degree of bioturbation (see also Ref.^27^). These ecological changes also coincide with a facies change representing a transgression and the development of a dominantly euxinic water column^27,28^. This faunal assemblage does not include any originally aragonitic body fossils, which means testing the H1 hypothesised acidification event at the onset of the mass extinction event is not possible using bioindicators.

A different faunal composition is recognised in the samples from 11.9 m and 12.6 m above base of section, which record a high species richness of bivalves, gastropods, ammonoids, brachiopods, foraminifera, radiolarians, and sponges. These assemblages correspond to the H2 ocean acidification interval during the *H. parvus* Zone. This biotic assemblage is unique for the Early Triassic as it is extremely well-preserved and records a high number of larval shells, enabling us to test for differences in the development of dissolution and repair marks between adult, juvenile, and larval stages. This is significant, because many of the species, e.g., *Nucinella taylori,* are interpreted to have had a planktotrophic larval stage and a benthic adult stage^25^. Thus, if ocean acidification only developed in the upper part of the water column, the dissolution and repair marks will be limited to the planktotrophic stages of shell growth. This assemblage is also associated with enhanced preservation and a facies change to shallower and locally oxic conditions.

### Dissolution scars

**Figure 2:**
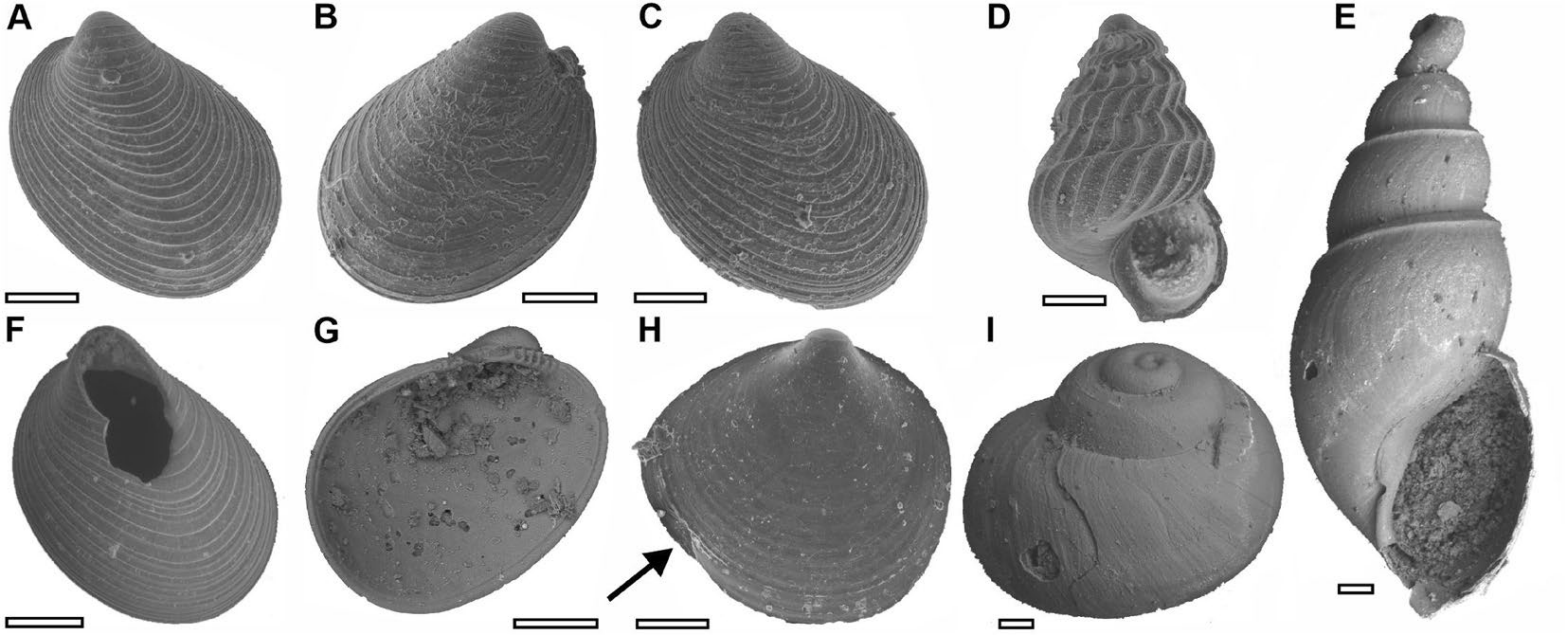
Scanning electron micrographs of representatives of the different states of shell preservation observed in this study. (**A**–**C**,**F**,**G**) *Nucinella taylori* Foster et al. (2017)^25^: (**A**) NHMUK PI MB 1339(29), pristine condition, (**B**,**C**) with dissolution marks, (**B**) NHMUK PI MB 1343(9), (**C**) NHMUK PI MB 1339(80), (**F**) NHMUK PI MB 1342(34), broken/damaged, (**G**) NHMUK PI MG 1587(5), dissolution marks on the internal side of the shell. (**D**) Pseudozgopleuridae indet., NHMUK PI MG 1581(7), pristine condition, (**E**) *Sinuarbullina yangouensis* (Pan & Erwin 2001), pristine condition. This specimen was lost when the material was accessioned. (**H**) Pectinidae? indet., NHMUK PI MB 1341(30), morphological deformity (arrow) inferred to have been caused by damage to the shell during growth. (**I**) *Glabrocingulum parvum* Foster et al. (2017)^25^*,* NHMUK PI MG 1520, small degree of damage. Scale bars = 100 µm.

**Figure 3:**
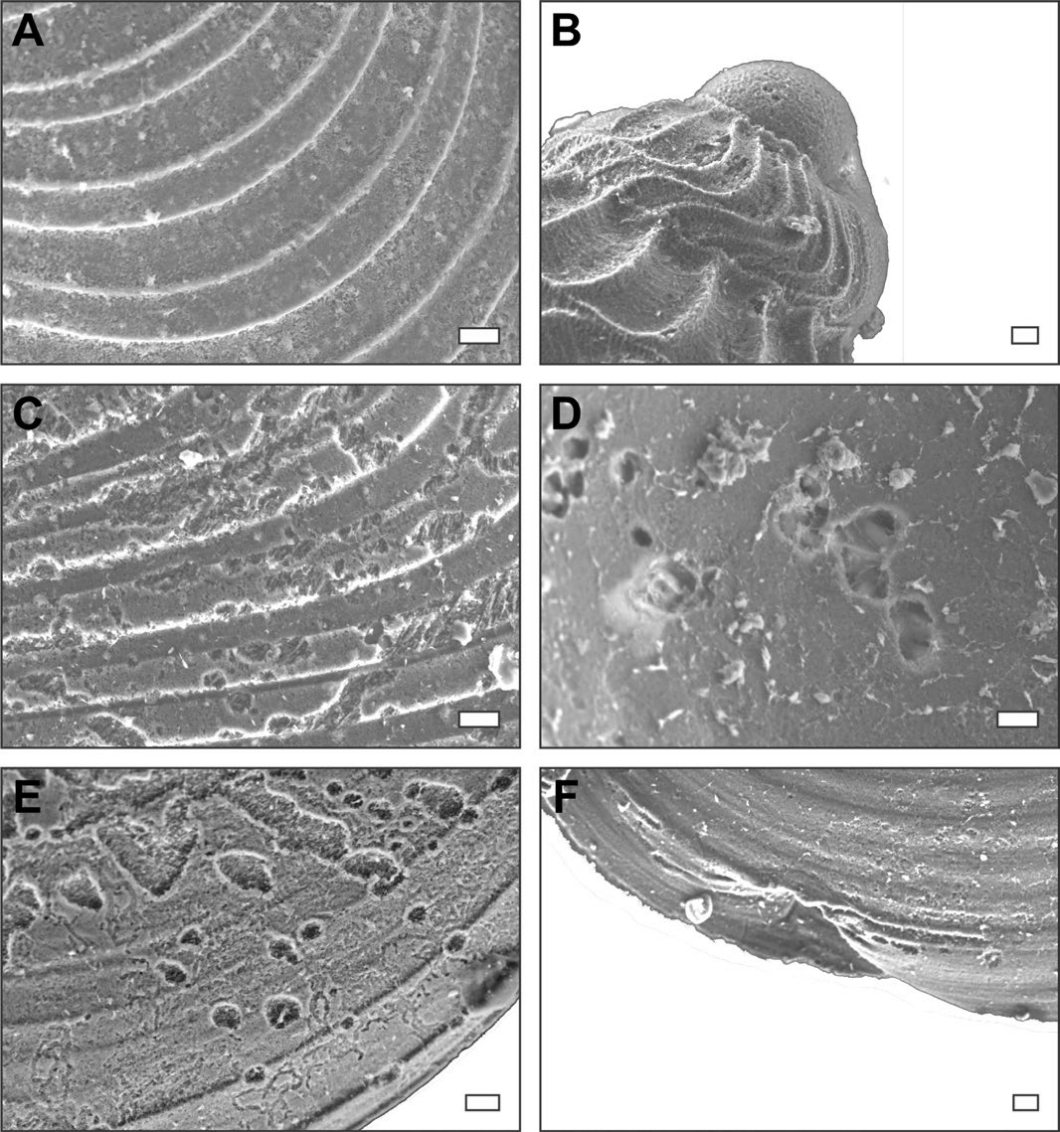
High magnification scanning electron micrographs of representatives of the different states of shell preservation observed in this study. (**A**,**C**–**E**) High magnification of *Nucinella taylori* Foster et al. (2017)^25^ shells. (**A**) the pristine shell in Fig. 2A. (**B**) Initial whorl of the Pseduozygopleuridae specimen in Fig. 2D. (**C**) The shell with dissolution pits in Fig. 2D. (**D**) Dissolution pits on the internal surface of the shell in Fig. 2G. (**E**) Dissolution pits and grooves of the shell in Fig. 2B. (**F**) Pectinidae? indet., morphological deformity inferred to have been caused by damage to the shell during growth (magnified from Fig. 2H). Scale bar = 10 µm.

**Figure 4:**
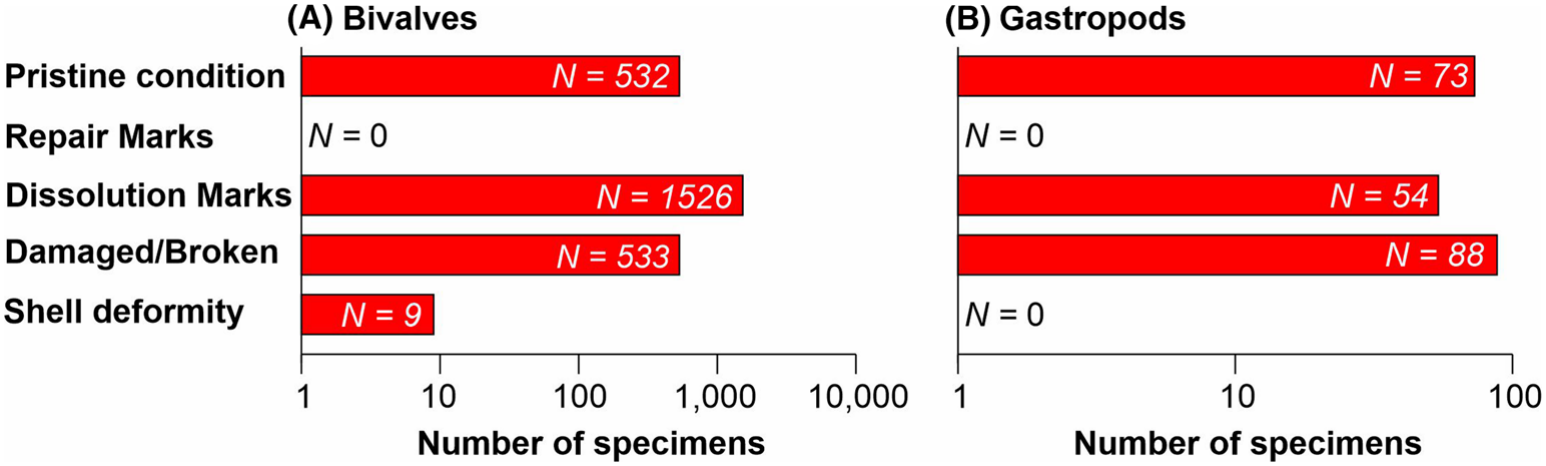
Bar charts showing the number of different shell preservation states for (**A**) bivalves and (**B**) gastropods. Note: the x-axis is logarithmic.

The original aragonitic shells of the bivalves and gastropods are replaced with either blackened calcite or silica. This investigation of shell deformities focussed on the specimens that were silicified and, based on the well-structured ultrastructure these shells, record excellent preservation with silica replacement (Supplementary Fig. S1). The bivalves and gastropods in this study do record dissolution marks and shell preservation can be characterised into different categories: (1) those with dissolution marks, (2) those that are fragmented, (3) those with growth deformities, and (4) those that do not record any damage (i.e., pristine) (Figs. 2, 3). Of the 2376 specimens investigated, 66% record dissolution marks, 26% are fragmented, < 0.1% record growth deformities, and 25% are pristine (Fig. 4). Fragmentation occurred post-mortem, and is an expected consequence of the shells being very thin (~ 10–50 µm thick) and the aggressive fossil extraction methods; damage was observed to occur whilst extracting the fossils from the disaggregated sediment. Dissolution marks, which occur in the form of pits and grooves across the shells, or large patches where the outer shell layer is missing (Figs. 2, 3), are recorded in most of the specimens but all are considered to be post-mortem, and none of the examples are inferred to be a consequence of ocean acidification. The dissolution marks are not associated with malformed growth lines or focussed at the apices or sutures of gastropods, which would be expected if dissolution had occurred whilst the animals were alive, i.e., the dissolution of shells did not lead to morphological deformation as has been observed in experimental studies (Figs. 2, 3). Some shells record similar dissolution scars exclusively on their internal surfaces only (e.g., Figs. 2G, 3D), supporting the interpretation that this dissolution occurred post-mortem. Crucially, none of the shells record evidence of any in vivo shell repair, which is a defence mechanism that molluscs utilise in acidified waters^19^, and which would be unequivocal evidence of severe acidification affecting the living animals. Only 9 fossils (< 0.1%) record evidence of in-life growth impairment (Figs. 2H, 3F, Supplementary Fig. S2). Furthermore, the presence of disordered fibres in the ultrastructure of bivalve shells have also been suggested as a bioindicator for ocean acidification^23^, and in this study none of the observed shell ultrastructures recorded disordered fibres. Therefore, the specimens investigated in this study do not record compelling evidence for an ocean acidification event during the *H. parvus* Zone.

## Discussion

### Shell calcification

Aragonite is a polymorph of calcium carbonate that is 50% more susceptible to dissolution than calcite in seawater^29^, which makes taxa that build their shells from aragonite more vulnerable to the effects of ocean acidification^30^. The end-Permian mass extinction event was, however, selective against taxa that build their shells from calcite, e.g., brachiopods and bryozoans, and most molluscs that survived the mass extinction constructed their shells using aragonite so the proportion of aragonitic taxa increased in the oceans^31^. A taxon’s vulnerability to ocean acidification not only depends on the calcium carbonate polymorph it uses to construct its skeleton, but also on its ability to regulate pH and carbonate chemistry at the site of calcification, how well the periostracum protects the shell, and the characteristics of the shell microstructure^30,32,33^. Even though many molluscs have adaptations that allow them to survive low pH and low saturation state scenarios, several studies have shown that they respond to ocean acidification in a few predictable ways that affect the calcification of the shell. These include changes in the shell thickness^18^, repair of the shell following dissolution^16,19,22,34–37^, reduced shell size^21,38^, shell deformation^18,19^, modified ultrastructure^18^, and shell weight^38,39^. This means that fossilised shells can be used to investigate ocean acidification in the past, at least where preservation allows.

This study investigated dissolution and shell deformities in exquisitely preserved fossil bivalve and gastropod species that possessed an aragonite shell^25^. Modern experiments and mesocosms (e.g., Ref.^19^), have demonstrated that if severe ocean acidification develops during the lifetime of an aragonitic mollusc, such as those studied here, it would leave a characteristic signature on the shells. In this study, although 66% of the molluscs investigated did record dissolution marks (Figs. 2, 3, 4), including dissolution pits, dissolution patches, and a network of dissolved grooves on the shell, no individuals recorded repair marks. The presence of dissolution features alone is not unequivocal evidence of ocean acidification. As has been shown with the modern *Limacina inflata* index, aragonite corrosion also occurs in the pore waters of the sediment after post-mortem burial, even if the overlying water column is not acidic^24^. Taphonomic studies have demonstrated that post-mortem dissolution and loss of aragonite within marine sediments and rocks is a widespread and common phenomenon affecting aragonitic molluscs throughout Earth history (e.g., Ref.^40^). Dissolution marks could be created at any stage of the post-mortem taphonomic process, including during collection and disaggregation of the samples. Therefore, it is critical to demonstrate that dissolution took place in the water column and whilst the animal was alive. Repair marks would be unequivocal evidence for severe in vivo acidification, but none of the shells investigated in this study record repair marks. Furthermore, dissolution marks also occur on the inside surfaces of the shells that would not have been exposed to the water column whilst the animal was alive, and, therefore, must have occurred after shell had been buried post-mortem. Fewer than 0.1% of the specimens record deformities, which indicate in vivo shell damage. These shell deformities could, however, have been caused by hydrological damage or attempted predation, and given that so few specimens record deformities they are unlikely to have been caused by severe ocean acidification.

Other predicted consequences of acidification that should be recognisable in the fossils include smaller shell sizes and changes in shell thickness^15^. It is noteworthy that the shells recovered are extremely thin (~ 10–50 µm thick) and are thus delicate, which led to mechanical damage during the preparation process (i.e., sieving and picking) and subsequently a relatively high fragmentation rate of 26% (Figs. 2, 3, 4). Nevertheless, it is not possible to determine if the thin shells are due to ocean acidification because some molluscs (as well as other calcifiers) respond to ocean acidification by producing thicker shells, e.g., *Patella caerulea*^18^. A reduction in the size of marine invertebrates through the mass extinction event is well documented^41^, and while a causal link between shell size decline and ocean acidification has been proposed^19^, this reduction in shell size could be caused by a number of abiotic changes, such as temperature, productivity, salinity and dissolved oxygen concentrations (e.g. Ref.^42^). Not all taxa that span the Permian/Triassic boundary show a size decrease (e.g., *Claraia liuqiaoensis* or *Unionites*), and the mollusc species recorded in this study are not known from pre-extinction sediments. The presence of thin and small shells in our specimens is, therefore, not considered a reliable or unequivocal indication of the presence of ocean acidification.

### Spatial and water depth variations

The best-known example of using mollusc-calcification changes to document changes in the aragonite saturation state is the development of the pteropod *Limacina inflata* index^43^. This index has also been applied to the fossil record, to investigate past aragonite saturation state dynamics (e.g., Refs.^24,44^). Aragonite is a relatively soluble form of biogenic calcium carbonate^29^ and the upper ocean is where the majority of atmospheric carbon dioxide is absorbed^45^. Although the pteropod fossil record does not extend beyond the Late Cretaceous^46^, marine gastropods with planktonic larvae evolved around the Cambrian-Ordovician transition^47^, and so their larval shells, preserved in the fossil record, provide a potential means of testing for ocean acidification in near-surface waters throughout most of the Phanerozoic.

An extraordinary finding of the bivalve and gastropod assemblages studied here is the excellent preservation of the early larval stages that record different ecological lifestyles. These fossils can provide information on the development of carbonate dissolution at a variety of water depths because the morphology of the bivalve and gastropod larval stages suggests that most of the benthic species had a planktotrophic larval stage^25^. It is the same planktotrophic trait that has also made pteropods an attractive bioindicator of ocean acidification in Cenozoic and modern-day studies (e.g., Ref.^24^). The shells of the bivalve *Nucinella taylori* are a characteristic and abundant example as they record the D-larval veliger stage (first phase of metamorphosis), change in shell shape and growth (second phase of metamorphosis), as well as the benthic adult stage. Functional morphological inferences suggest that most of the bivalves and gastropods in this study would have had a similar planktotrophic to benthic life history^25^. Since only 9 bivalve larval shells (< 0.1%) recorded morphological deformities in the benthic stage (e.g., Fig. 3F), it is likely that no parts of the water column experienced severe ocean acidification (or at least not to levels that were detrimental to these aragonitic invertebrates) during the deposition of these fossiliferous horizons in the earliest Triassic *H. parvus* Zone.

A key concept that is often overlooked when investigating ocean acidification during past climate crises, especially during Permian–Triassic and Triassic–Jurassic extinction intervals, is that the aragonite saturation state of the world’s oceans is spatially variable. Furthermore, the degree of change as a consequence of a carbon injection also varies spatially^48^. Where the largest projected decreases in aragonite saturation are predicted in warm tropical and subtropical waters^48^. The hypothesis of an ocean acidification event during the *H. parvus* Zone comes from an interpretation of the δ^44/40^Ca signature of bulk rock samples and conodont elements from a substantial number of shallow, palaeotropical localities^9–11^. The actual timing of this geochemical excursion varies slightly between sections, owing to issues with the precision of stratigraphic correlations, but generally the δ^44/40^Ca record has been used to infer a basal Triassic ocean acidification event^11^. The Permian–Triassic δ^44/40^Ca record has not been investigated in Svalbard so the relationship between the presence/absence of dissolution and repair marks on molluscs and the δ^44/40^Ca signature locally is unknown. In addition, Svalbard’s palaeogeographic location means it was located in the temperate climate of the Boreal Ocean and may have experienced less severe ocean acidification than subtropical and tropical settings. Whilst our results enable us to reject the hypothesis that a worldwide ocean acidification event took place during the *H. parvus* Zone (i.e., affecting every location studied), they only represent a regional proxy, and we are thus cautious in extrapolating to other regions during this interval using the Svalbard data alone.

**Figure 5:**
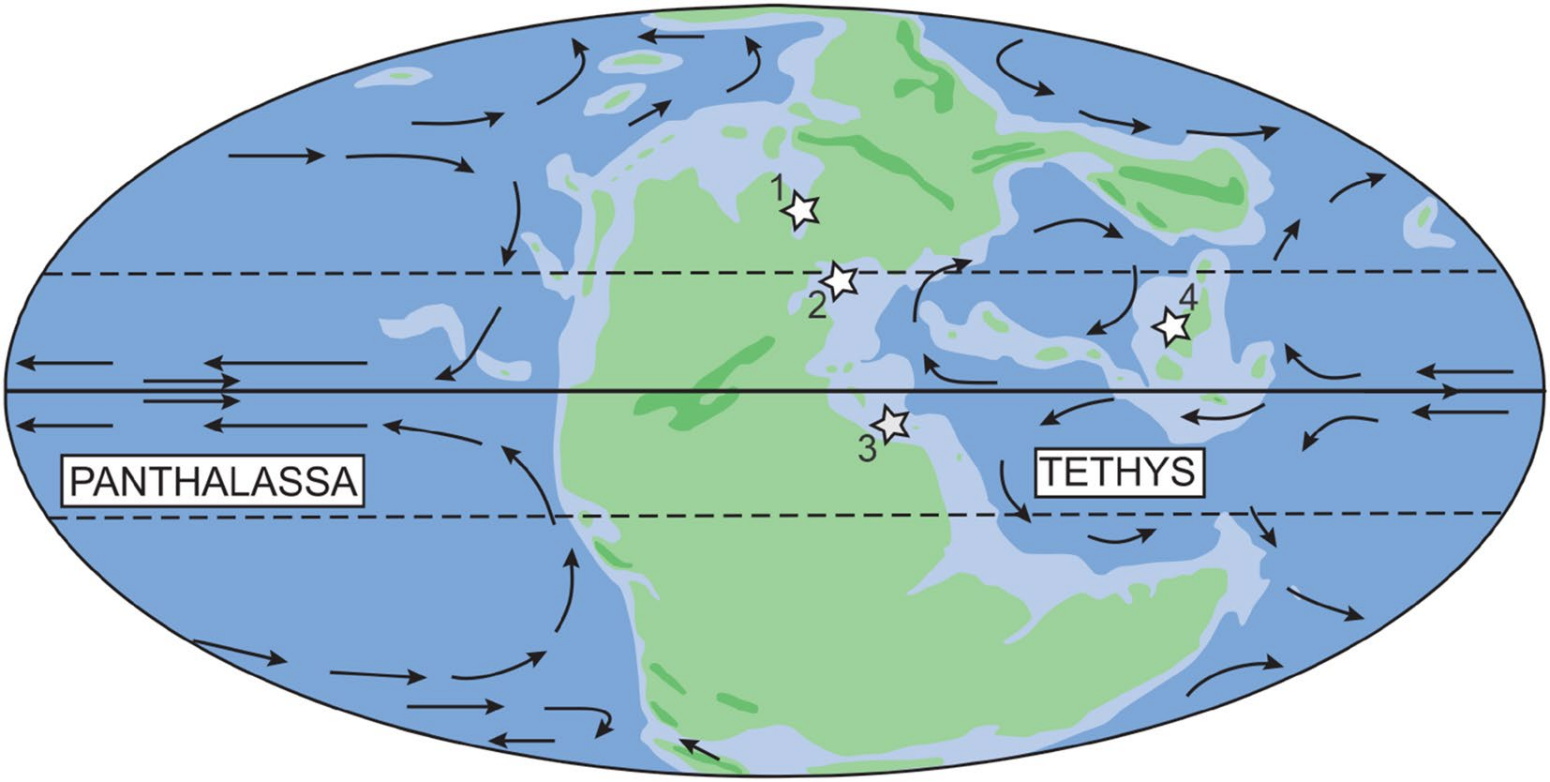
Palaeogeographic map showing the location of (1) Svalbard, (2) Italy & Hungary, (3) Oman, and (4) South China during the Permian/Triassic boundary. Base map from Golonka^58^ and interpreted oceanic currents from Flügel^59^.

A review of systematic descriptions and figures of bivalves and gastropods recorded from the Permian/Triassic boundary shows that bioindicators of severe ocean acidification are also absent from other regions (Fig. 5; Supplementary Table S2), supporting the data from Svalbard. In the Dolomites (Italy) and Bükk Mountains (Hungary), poorly-preserved bivalves and gastropods are recorded from both post-extinction Permian and basal Triassic strata^49,50^. None of the described or figured specimens record bioindicators consistent with severe ocean acidification. In partially silicified assemblages from Oman^51,52^, the apices of high-spired gastropods are intact, which would not be expected if they experienced severe ocean acidification. Examples where protoconchs are missing and shell apertures are not fully preserved are most likely the result of the coarse silicification^51,52^ and not ocean acidification. Bioindicators of severe ocean acidification are, therefore, absent in Oman too. In South China, well-preserved molluscs also do not record dissolution and repair marks or growth deformities consistent with ocean acidification^53–55^. Moreover, these fossil assemblages described from Italy and South China were recovered from the same interval as the δ^44/40^Ca excursion, and from anachronistic carbonate facies (e.g., Ref.^11^). Together, the lack of bioindicators of severe ocean acidification from multiple regions, including in tropical localities, suggests that it is implausible that there was an ocean acidification event during the basal Triassic *H. parvus* Zone.

## Conclusion

Here, we used a well-preserved fossil mollusc fauna to test predictions concerning the presence and timing of two supposed ocean acidification events through the Permian–Triassic interval. During the hypothesised latest Permian ocean acidification event, which is associated with the extinction itself, the recorded fauna comprised siliceous sponges and organophosphatic brachiopods. The absence of preserved molluscs meant that the acidification hypothesis could not be tested for this older event. The absence of preserved molluscs is likely due to post-mortem dissolution, which is a common phenomenon affecting the fossil record throughout the Phanerozoic and is unrelated to ocean acidification. A recorded reduction in the size of the brachiopods is consistent with acidification but also with other environmental changes (e.g., oxygen stress), and so does not represent an unequivocal test of ocean acidification. During the hypothesised earliest Triassic *H. parvus* Zone ocean acidification event, however, the recorded fauna is very diverse and includes aragonitic molluscs which can be used to test the ocean acidification hypothesis. None of the specimens recorded the characteristic repair marks that would indicate in vivo acidification and < 0.1% of the specimens recorded morphological deformities that would be expected in an ocean acidification scenario. Surface dissolution of the shells is present but occurred post-mortem, probably due to corrosion by porewaters post-burial. These results enable us to reject the hypothesis of a basal Triassic ocean acidification event, at least in Svalbard, as postulated from calcium isotopes. Reports of well-preserved molluscs from *H. parvus* Zone fossil assemblages in other locations worldwide suggests that climate-driven acidification, if present at all, would have been local at best and did not affect the whole ocean.

## Materials and methods

### Geological setting

Samples investigated in this study were collected from the Permian–Triassic succession at Lusitaniadalen, central Svalbard (78° 17′ 54.82″ N, 016° 43′ 59.3″ E) (Fig. 1). The Permian–Triassic succession represents deposition on an open-marine, shelf setting^56^, in a temperate climate around 45–50° N in the southern part of the Boreal Ocean^56^. At Lusitaniadalen, the basal 1.6 m of the Vikinghøgda Formation (Deltadalen Member) comprises of bedded, glauconitic sandstones that can be distinguished from the underlying Kapp Starostin Formation by the absence of chert^56^. These sandstones are well-bioturbated by a diverse suite of trace fossils that have been used to indicate a fully functioning benthic ecosystem prior to the mass extinction event^27^. The sudden loss of this diverse infauna occurs just prior to the top of the sandstones and marks the local collapse of marine ecosystems during the mass extinction event^27^. The top of the sandstones is a transgressive surface, which is overlain by c. 10 m of laminated mudstones. In the lower 2 m of the laminated mudstone interval, fine-grained, pyritic and glauconitic, graded, cemented sandstones, interpreted as distal tempestites, also occur. In the nearby Deltadalen section, Zuchuat et al.^26^ recorded the conodont *Hindeodus parvus* from one of these concretionary horizons 2.4 m above the top of the glauconitic sandstones, which marks the Permian/Triassic boundary. Both lipid biomarkers and Fe speciation data suggest that deposition was in a dominantly dysoxic–euxinic environment^27,28^. Above the laminated mudstones, the rocks have an increasing proportion of interbedded siltstones and very-fine sandstones, reflecting shelf progradation^56^. There is also an increase in the degree and depth of bioturbation, which indicates deposition in a more oxygenated setting^27^. A thick concretionary, very fine sandstone bed at 12.6 m above the base of the Vikinghøgda Formation records the ammonoid *Otoceras boreale* and the conodont *Neogondolella carinata,* which indicate an early Griesbachian age^56^.

### Sampling protocol, preparation, and analysis

250 g samples were collected every 50 cm throughout the succession from all the lithologies. Each sample was then disaggregated using 10% hydrogen peroxide (H_2_O_2_) with the solution changed every 48 h. In addition, 3 kg samples were collected from each concretionary horizon. The samples from the concretionary horizons were mechanically disaggregated into 1–2 cm-sized blocks, and the pieces that did not have fossils on their surfaces were disaggregated with the buffered formic acid technique^57^. The samples were sieved every 6–12 h and dried in a 40 °C oven. The fossils were separated from the disaggregated sediment using a binocular microscope and wet paintbrush. Each fossil specimen was then imaged using a scanning electron microscope at the University of Texas at Austin and University College Dublin. The quality of the preservation was investigated for each specimen and the presence/absence of the following features were noted for each shell: (1) no dissolution or deformities (i.e., pristine), (2) dissolution marks including pits, patches, and grooves, (3) repair marks, (4) morphological abnormalities, and (5) fragmentation (Fig. 2). Morphological abnormalities were distinguished as either malformations (i.e., abnormal development) or deformations (i.e., mechanical damage to the shell).

The specimens are housed in the Natural History Museum, London, United Kingdom (NHMUK).

## Supplementary Information

Supplementary Figures.

Supplementary Table S1.

Supplementary Table S2.

Supplementary Table S3.

## Data Availability

All data are available in the main text or the supplementary materials.
