# Supplementary material for: Bioindicators of severe ocean acidification are absent from the end-Permian mass extinction
Source: Sci Rep. 2022 Jan 24;12:1202. doi: 10.1038/s41598-022-04991-9 (PMC8786885; doi:10.1038/s41598-022-04991-9)
Supplement: Supplementary file 1 — Supplementary Figures. [file 41598_2022_4991_MOESM1_ESM.docx]

Supplementary Figures for

**Bioindicators of severe ocean acidification are absent from the end-Permian mass extinction**

W. J. Foster,^*^ J. Hirtz, C. Farrell, M. Reistroffer, R. J. Twitchett, R. C. Martindale,

*Corresponding author. Email: [william.foster@gmx.co.uk](mailto:william.foster@gmx.co.uk)


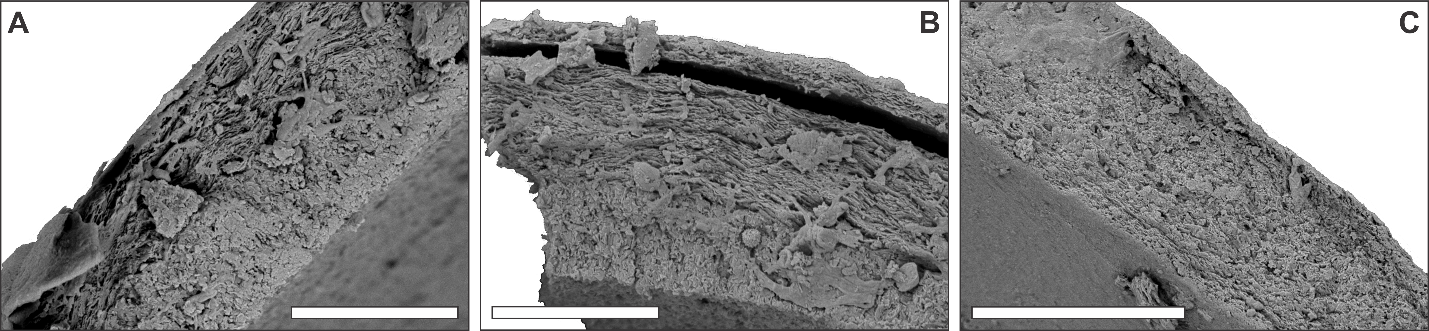


Fig. S1. Scanning electron micrographs of fragmented surfaces of silicified molluscs from Lusitaniadalen, Svalbard, showing the shell ultrastructure. (A) *Warthia zakharovi*, (B) *Nucinella*? (A-B) Scale bar = 30µm (C) *Austrotindaria svalbardensis*, scale bar = 50µm.


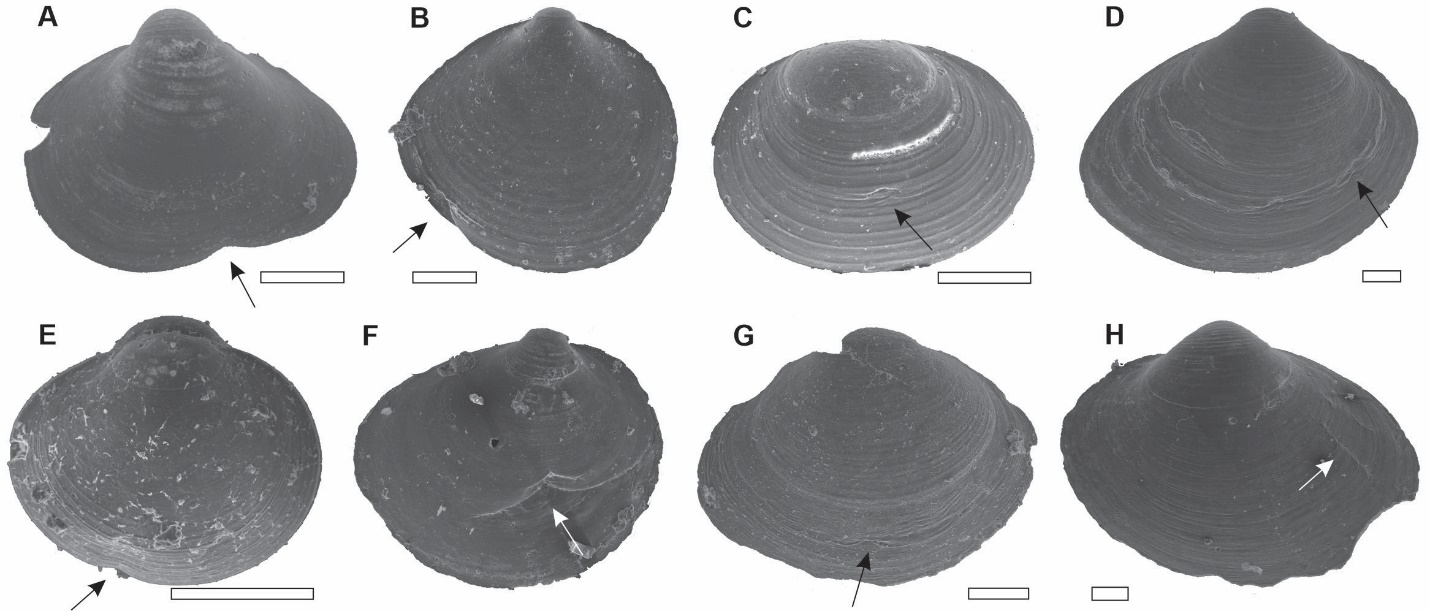


Fig. S2. Scanning electron micrographs of all the specimens that show shell deformities, indicated by an arrow. (A) *Pteria* sp., NHMUK PI MB 1369(6), (B) *Neoschizodus* *laevigatus*, NHMUK PI MB 1341(30), (C) Mallettidae sp., NHMUK PI MB 1386(36), (D) *Austrotindaria* *svalbardensis*, NHMUK PI MB 1369(25), (E) *Neoschizodus* *laevigatus*, NHMUK PI MB 1351(4), (F) *Neoschizodus* *laevigatus*, NHMUK PI MB 1362(36), (G) *Austrotindaria* *svalbardensis*, NHMUK PI MB 1369(26), (H) *Austrotindaria* *svalbardensis,* NHMUK PI MB 1389(7). Scale bar = 100µm.
