# Supplementary material for: Bioindicators of severe ocean acidification are absent from the end-Permian mass extinction
Source: Sci Rep. 2022 Jan 24;12:1202. doi: 10.1038/s41598-022-04991-9 (PMC8786885; doi:10.1038/s41598-022-04991-9)
Supplement: Supplementary file 2 — Supplementary Table S1. [file 41598_2022_4991_MOESM2_ESM.docx]

**Table S1: Descriptions of dissolution and repair marks caused by ocean acidification, hydrological damage, and predation.**

| **Ocean acidification** |
| --- |
| Ocean acidification can lead to many different forms of morphological damage to the shells of bivalves and gastropods:  Generally, dissolution is focused on the oldest part of the shells: in gastropods this can lead to the truncation of the shell, i.e., severe dissolution and subsequent loss of the apex (Garilli et al., 2015; Harvey et al., 2018). In gastropods that survive such apical dissolution, the truncated apex is repaired with a calcareous plug and the shell remains truncated (Garilli et al., 2015).  Dissolution can also lead to fractures of the periostracum, which are followed by dissolution of the shell mineral layers. This dissolution forms irregular fissures and/or pits (Tunnicliffe et al., 2009; Garilli et al., 2015). In gastropods, this dissolution can be focused on the sutural area prior to shell truncation (Garilli et al., 2015). In bivalves, irregularly-shaped small to large patches of dissolution may affect all shell layers (Tunnicliffe et al., 2009). This *in vivo* dissolution is external and so may be readily distinguished from post-mortem dissolution which may occur on both the internal and external surfaces of the shell.  In bivalves (and articulated brachiopods) dissolution can also lead to notches on the outer shell, which may or may not affect subsequent growth (Cross et al., 2015). In cases where they do not affect shell growth, these notches are preserved in the profile of the growth lines. Where these notches do affect shell growth, subsequent growth deformations are recorded (Bylenga et al., 2017). In extreme cases, ocean acidification can lead to uncalcified organisms that are preserved as moulds of the organism (Bylenga et al., 2017), but this is not distinguishable from diagenesis in the rock record. |
| **Hydrological damage** |
| The shells of bivalves and gastropods are known to be affected by hydrological damage in the fossil record. This includes the erosion of umboes/apices, abrasion causing small areas of concentrated pits or fragmentation, or abrasion during post-mortem transport which causes the shells to become rounder clasts (Kotzian and Simões, 2006). Hydrological damage is expected to be more prominent in environmental settings affected by storms, fast water currents, water flow associated with river discharge, and abrasive substrates (Kotzian and Simões, 2006).  The environmental setting of Lusitaniadalen suggests that deposition was in a low-energy setting below wave base that is unlikely to have experienced fast-moving water that would have abraded benthic invertebrates. These fossils from Svalbard also do not show any signs of bioencrustation suggesting that they were rapidly buried and not exposed on the seafloor long enough for secondary tierers to encrust the shells, which is common in Early Triassic benthic communities (Fraiser, 2011). |
| **Predation shell damage** |
| Shell damage caused by predation on benthic invertebrates comprises three specific morphologies that can be easily distinguished from damage caused by ocean acidification:  1) Drill holes: Following Hoffmeister et al. (2004), drill holes are “(1) circular or oval, and [often] unhealed; (2) perpendicular to the shel; (3) penetrate only one valve of articulated specimens; and (4) likely penetrate the valve from the outside (i.e., the outer hole diameter exceeds the inner hole diameter).” In addition, drill holes only tend to occur in specific positions on the shell and usually only one successful hole is present on the shell of any one individual.  Not all drill holes identified in the shells of bivalves and gastropods could be a result of predation, as some drill holes may actually result from other biological activity, such as boring into the substrate (Richards and Shabica, 1969). However, in our study of faunas from Lusitaniadalen, any boring ecological lifestyles would have been limited to wood-fall communities and not the assemblage of soft-substrate molluscs that was investigated.  2) Inferred vertebrate bite marks: Damage to the shell includes a series of regularly  spaced indentations with associated depression fracturing and some surface crushing (Kear et al., 2008). Durophagous predators are likely to have completed crushed the shells (Stafford and Leighton, 2011).  3) Peel marks: these are generally restricted to gastropods and form where the predator (typically an arthropod) has “peeled” back the shell from the aperture. Damage is usually confined to the last whorl, or indicated by the presence of a broken piece of the aperture. Gastropods may survive such an attack and subsequently repair their shell. In these circumstances the repair would be restricted to the last whorl where the aperture was damaged, and depending on the severity of the attack subsequent growth may be malformed (Kowalewski, 2002). |
|  |
|  |

**References**

Bylenga, C.H. Cummings, V.J. Ryan, K.G. High resolution microscopy reveals significant impacts of ocean acidification and warming on larval shell development in *Laternula* *elliptica*. *PloS one* **12**, e0175706 (2017).

Cross, E. Peck, L.S. Harper, E.M. Ocean acidification does not impact shell growth or repair of the Antarctic brachiopod *Liothyrella uva* (Broderip, 1833). *Journal of Experimental Marine Biology and Ecology* **462**, 29-35 (2015).

Fraiser, M. L. Paleoecology of secondary tierers from Western Pangean tropical marine environments during the aftermath of the end-Permian mass extinction. *Palaeogeography, Palaeoclimatology, Palaeoecology* **308**, 181-189 (2011).

Garilli, V. Rodolfo-Metalpa, R. Scuderi, D. Brusca, L. Parrinello, D. Rastrick, S.P.S et al. Physiological advantages of dwarfing in surviving extinctions in high-CO_2_ oceans. *Nature Climate Change* **5**, 678-683 (2015).

Hoffmeister, A.P., Kowalewski, M. Baumiller, T.K. Bambach, R. K. Drilling predation on Permian brachiopods and bivalves from the Glass Mountains, west Texas. *Acta Palaeontologica Polonica* **49**, 443-454 (2004).

Kear, Benjamin P., and Henk Godthelp. Inferred vertebrate bite marks on an Early Cretaceous unionoid bivalve from Lightning Ridge, New South Wales, Australia. *Alcheringa* **32,** 65-71 (2008).

Kotzian, C.B. Simões, M.G. Taphonomy of recent freshwater molluscan death assemblages, Touro Passo Stream, Southern Brazil. *Revista Brasileira de Paleontologia* **9**, 243-260 (2006).

Kowaleski, M. The fossil record of predation: An overview of analytical methods. *The Paleontological Society Papers* **8**: 3-42 (2002).

Richards, R.P. and Shabica, C.W. Cylindrical living burrows in Ordovician dalmanellid brachiopod beds. *Journal of Paleontology* **43**: 838–841 (1969).

Stafford, E.S.,and Leighton, L.R. Vermeij crushing analysis: a new old technique for estimating crushing predation in gastropod assemblages. *Palaeogeography, Palaeoclimatology, Palaeoecology* **305**: 123-137 (2011).

Tunnicliffe, V. Davies, K.T.A. Butterfield, D.A. Embley, R.W. Rose, J.M. Chadwick, W.W. Survival of mussels in extremely acidic waters on a submarine volcano. *Nature Geoscience* **2**, 344-348 (2009).
